# Extubation timing and risk of extubation failure in aneurysmal subarachnoid hemorrhage patients

**DOI:** 10.1186/s41016-024-00384-1

**Published:** 2024-11-20

**Authors:** Jun Yang, Junlin Lu, Runting Li, Fa Lin, Yu Chen, Heze Han, Ruinan Li, Zhipeng Li, Haibin Zhang, Kexin Yuan, Hongliang Li, Linlin Zhang, Guangzhi Shi, Shuo Wang, Xiaolin Chen

**Affiliations:** 1 Department of Critical Care Medicine, Beijing Tiantan Hospital, Capital Medical University, Beijing, China; 2 Department of Neurosurgery, West China Hospital, Sichuan University, Chengdu, China; 3 Department of Neurosurgery, Beijing Tiantan Hospital, Capital Medical University, No.119 South 4th Ring West Road, Fengtai District, Beijing, 100070 China; 4 China National Clinical Research Center for Neurological Diseases, Beijing, China; 5 Center of Stroke, Beijing Institute for Brain Disorders, Beijing, China; 6 Beijing Key Laboratory of Translational Medicine for Cerebrovascular Disease, Beijing, China

**Keywords:** Aneurysmal subarachnoid hemorrhage, Extubation failure, Delayed cerebral ischemia, Microsurgery

## Abstract

**Background:**

The extubation time is critical during the intensive care unit stay in aneurysmal subarachnoid hemorrhage (aSAH) patients. The current conventional parameters for predicting extubation failure (EF) and extubation time may not be suitable for this population. Here, we aimed to identify factors associated with EF in aSAH patients.

**Methods:**

From a single-center observational study on aSAH patients with computed tomography angiography from 2019 to 2021, patients who received microsurgery were enrolled and divided into two groups according to whether EF occurred. Multivariable logistic regression was conducted to evaluate disease severity, medical history, and extubation time differences between patients with and without EF.

**Results:**

Of 335 patients included, EF occurred with a rate of 0.14. Delayed cerebral ischemia (67.4% vs. 13.5%) and acute hydrocephalus (6.5% vs. 1.4%) were frequently observed in patients with EF. Also, patients who develop EF presented higher disability (65.9% vs. 17.4%) and mortality (10.9% vs. 0.7%) rates. Multivariable analysis demonstrated that age (OR 1.038; 95% CI 1.004–1.073; *P* = 0.028), onset to admission time (OR 0.731; 95% CI 0.566–0.943; *p* = 0.016), WFNS grade > 3 (OR 4.309; 95% CI 1.639–11.330; *p* = 0.003), and extubation time < 24 h (OR 0.097; 95% CI 0.024–0.396; *p* = 0.001) were significantly associated with EF occurrence.

**Conclusions:**

These data provide further evidence that older aSAH patients with onset to admission time < 2 days and WFNS grade > 3 have a high risk of developing EF, which is amplified by the ultra-early extubation. Moreover, in patients with two or more risk factors, a prolonged intubation recommendation requires consideration to avoid the EF.

**Supplementary Information:**

The online version contains supplementary material available at 10.1186/s41016-024-00384-1.

## Background

Aneurysmal subarachnoid hemorrhage (aSAH) constitutes a life-threatening subtype of stroke affecting patients at a mean age of 55 years, leading loss of many years of productive life, accounting for more than 85% of all subarachnoid hemorrhage without previous trauma [1]. It is a neurological emergency that requires prompt diagnosis and management to prevent life-threatening rebleeding and optimize patient outcomes [2]. Better diagnosis, early repair of the aneurysm, and advanced intensive care support increased survival from aSAH in recent decades [3]. Patients with aSAH often need intubation, mechanical ventilation, and consecutive tracheotomy after microsurgical clipping treatment, especially with poor Hunt and Hess (H–H) grade [4].

Patients after microsurgical treatment often require intubation for airway protection to avoid respiratory failure. The need for prolonged intubation due to the severity of brain injury, respiratory failure, and other organ dysfunction prolongs the stay in the ICU and is associated with higher mortality [5]. The day of extubation is critical during the intensive care unit (ICU) stay in aSAH patients who survive an episode of intubation with mechanical ventilation. Extubation is usually decided after a weaning readiness test that involves either spontaneous breathing on a T-piece or a low level of ventilatory assistance [6]. However, extubation failure (EF) still occurs in some patients even after a thorough evaluation. EF is usually defined as the need for reintubation within hours or days following extubation. However, the time interval used in the definition varies from 48 to 72 h or 1 week. Generally, EF occurs in 10 to 20% of patients and is associated with extremely poor outcomes, including high mortality rates of 25 to 50% of patients in the ICU [6–8]. Since available studies demonstrated that neurocritical patients show a high range (5 ~ 40%) of EF rates [4], and several risk factors have been proven to predict EF, it remains uncertain whether these predictors can be used in aSAH patients. Also, there is a lack of recommendations for time interval extubation in aSAH patients who received microsurgical clipping.

In our center, we have 2 departments treating aSAH patients with endovascular coiling and surgical clipping. The International subarachnoid aneurysm trial (ISAT) study and current guidelines suggest that if the patient can be treated with both methods, endovascular therapy is preferred [9]. Although endovascular treatment has advantages over surgical clipping in terms of discharge and 90-day outcomes, and in-hospital complications [10]. The data we analyzed came from the microsurgical clipping group in this article.

Thus, in the current observational cohort study, we aimed to identify the early occurring factors associated with EF in microsurgical clipping aSAH patients. Furthermore, we investigated the relationship between EF and prognosis in the study population. This information helps clinicians optimize decision-making concerning the right timing of extubation.

## Methods

### Study design, setting, and participants

We retrospectively reviewed patients diagnosed with aSAH and received microsurgical clipping in our department from December 2019 to January 2021. All patient data were from the Long-term Prognosis of Emergency Aneurysmal Subarachnoid Hemorrhage (LongTEAM) study. The registry is listed at ClinicalTrials.gov (registration no. NCT 04785976). The local institutional review boards (IRBs) of all participating centers of the prospective registry approved the study with a waiver of consent in December 2019. Secondary use of the registry data and additional review of medical records for this study were approved by IRBs. All the analyses were performed in accordance with the Declaration of Helsinki and the local ethics policies. The study was approved by the Beijing Tiantan Hospital Research Ethics Committee. Informed consent was obtained from the patient or the surrogate decision-maker before enrollment.

In this study, the inclusion criteria were (1) patients who were diagnosed with aSAH by computed tomography (CT) and digital subtraction angiography or CT angiography, (2) adult patients (> 18 years of age), (3) aneurysms were treated by microsurgery clipping, (4) subarachnoid hemorrhage diagnosed to treated by microsurgery less than 7 days. Patients combined with congenital cerebral vascular disease (e.g., arteriovenous malformations and moyamoya disease) and with coexistent intracranial lesions were simultaneously treated (e.g., resection of meningioma or pituitary adenoma) were excluded from the present study.

### Perioperative management

Patient management conformed to guidelines set forth by the American Heart Association [11]. All patients had angiographically documented aSAH confirmed by CT or lumbar puncture. During the operation, all patients were intubated, mechanically ventilated, and general anesthesia as well as for muscle relaxation. We routinely transferred the patients to the ICU and retained the endotracheal intubation after the microsurgery. To determine the timing of extubation, we used conventional pulmonary weaning parameters adapted from our pre-existing weaning protocol with the following criteria: (1) sufficient spontaneous breathing trial using pressure support ventilation or continuous positive airway pressure ventilation with a respiratory rate < 30, minute volume ≤ 10 L/min, tidal volume ≥ 5 mL/kg, positive end-expiratory pressure of < 8 cm H_2_O; (2) constant eye contact and obeying commands, including a Glasgow Coma Scale > 10; (3) extubation was performed based on an individual decision at the discretion of the treating neurointensivist, especially in patients with decreased levels of consciousness; (4) stable hemodynamic status without the use of inotropic agents or vasopressors, stable metabolic status, and weaning underwent at least 30 min. Nevertheless, in 10–20% of patients who pass a spontaneous breathing trial and undergo planned extubation, extubation failure still occurs [12]. Great care also was taken in known pulmonary or heart diseases before extubation in case of the influence of these factors [13]. A higher H–H grade implies a decreased level of consciousness and focal neurologic deficits resulting in inadequate protection reflexes and risk for aspiration. Finally, extubation was performed based on an individual decision at the discretion of the treating neurointensivist, especially in patients with decreased levels of consciousness [14].

### Variables, data source, and measurements

As part of an ongoing prospective cohort study, baseline characteristics of patients, radiological information, and hospital complications were collected, including age, sex, features of the aneurysm, and medical and medication history. Clinical grading was done with the use of the H–H grade and World Federation of Neurosurgical Societies (WFNS) grade at the initial presentation [15]. Admission CT scans were scored using the modified Fisher Scale (mFS) with 3–4 points classified as poor-grade mFS. Patients exhibited evidence of (1) CT presence of midline shift > 5 mm or obliteration of perimesencephalic cisterns; (2) dilation of pupils that were unresponsive to light, unilaterally or bilaterally [16, 17]; and (3) presence of subfalcine, transtentorial, or tonsillar herniation were defined as brain herniation. The size of the aneurysm was measured as its maximal diameter, and the location of the aneurysm was grouped according to its parent artery. Postoperative clinical complications during hospitalization were collected, including EF, delayed cerebral ischemia (DCI), and hydrocephalus. DCI was defined as clinical deterioration, the occurrence of a new focal neurologic deficit or a new infarction on CT that is not attributable to other causes [18]. We evaluated the patients’ prognosis by modified Rankin Score (mRS) at hospital discharge. The period from the microsurgery to extubation was recorded and defined as the time of extubation. Furthermore, we categorized the time extubation into two groups, the ultra-early (< 24 h) and early (≥ 24 h) groups. A needed reintubation within hours or days after the planned extubation was defined as EF.

### Quantitative variables and statistical methods

All statistical analyses were performed with SPSS Statistics 26.0 (IBM, Armonk, NY, USA) and GraphPad PRISM 9.0 (GraphPad Software Inc., San Diego, CA, USA). Statistical significance was established at *p* values < 0.05 for 95% confidence intervals (CI).

This is a retrospective analysis of prospectively recorded data. All consecutive patients included in our prospective cohort who met the inclusion criteria were included in the final analysis. No sample size calculation was performed. Categorical variables are summarized as frequencies (percentages) and continuous variables as mean ± SD or median (IQR). With the use of univariate analysis (*t*-test, Mann–Whitney *U* test, Pearson chi-square test, continuity correction test, or Fisher exact test, as appropriate), we identified clinically significant variables associated with EF. To estimate the association between baseline variables and EF occurrence, a multivariable logistic regression model (forward stepwise) was constructed using clinically relevant variables or with univariate *p* values < 0.15. The odds ratios (OR) and 95% CI of the variables were calculated.

To investigate the optimal extubation time in aSAH patients with microsurgery, a propensity score matching (PSM) was carried out to adjust for potential baseline confounding characteristics when comparing in-hospital complications and outcomes between ultra-early and early extubation groups. Patients in the ultra-early and early extubation groups were matched 1:1 with a caliper of 0.1 and without replacement. Standardized mean differences of included variables in the raw dataset and matched dataset were calculated to verify the efficacy of PS matching. The McNemar test was used to compare in-hospital complications and outcomes in the matched pairs. To further determine the effect of extubation time in different subgroups of aSAH patients, we conducted a subgroup analysis by risk factors for EF.

We computed a prediction equation for EF in aSAH patients based on each risk factor’s β coefficients in the multivariate analysis, referring to the following Eq. (1):

1 $$P=\frac1{1-e^{-LP}}$$

with LP = a + b1X1 + b2X2 + … + bpXp, b1, b2,…, bp as the regression coefficients associated with each of the variables in the final logistic model. To facilitate practical application of the model, we used the regression coefficients of the predictors in the final model, to allocate points to each predictor to generate a risk score. We translated the regression model into a score chart by dividing all regression coefficients by the smallest coefficient and subsequently rounded them to the nearest integer.

## Results

### Patient and lesion characteristics

A total of 335 aSAH patients harboring 397 aneurysms were identified. Detailed information on demographics, hospital complications, and outcomes is given in Table 1. The patients had an average age of 53.8 ± 10.3 years. The majority of patients received microsurgery within 3 days after the aneurysm ruptured. Most patients (277/335, 82.7%) had a mild neurological deficit on admission, even if 34.9% (117/335) patients had a mFS of 4.

**Table 1:** Baseline characteristics, complications, and outcome

	Total	EF	None-EF	*p* value
Characteristics	*n* = 335	*n* = 46	*n* = 289	
Age, years	53.8 ± 10.3	56.0 ± 9.4	53.5 ± 10.5	0.588
Sex				0.727
Female	190 (56.7)	25 (54.3)	165 (57.1)	
Male	145 (43.3)	21 (45.7)	124 (42.9)	
Comorbidities				
Smoking	59 (17.6)	9 (19.6)	50 (17.3)	0.708
Drinking	31 (9.3)	7 (15.2)	24 (8.3)	0.133
Hypertension	156 (46.6)	26 (56.5)	130 (45.0)	0.145
Diabetes	24 (7.2)	4 (8.7)	20 (6.9)	0.665
Hyperlipemia	9 (2.7)	3 (6.5)	6 (2.1)	0.083
Respiratory disease	4 (1.2)	1 (2.2)	3 (1.0)	0.510
Coronary disease	10 (3.0)	2 (4.3)	8 (2.8)	0.559
Prior infarction	22 (6.6)	5 (10.9)	17 (5.9)	0.205
Prior hemorrhage	2 (0.6)	1 (2.2)	1 (0.3)	0.135
Admission characteristics				
Onset to admission (days)	2 (1–3)	1 (0–2)	2 (0–4)	0.005
Brain herniation	10 (3.0)	2 (4.3)	8 (2.8)	0.559
H–H grade				0.01
1	40 (11.9)	3 (6.5)	37 (12.8)	
2	193 (57.6)	24 (52.2)	169 (58.5)	
3	5 (1.5)	3 (6.5)	2 (0.7)	
4	97 (29.0)	16 (34.8)	81 (28.0)	
WFNS grade				< 0.001
1	142 (42.4)	8 (17.4)	134 (46.4)	
2	135 (40.3)	23 (50.0)	112 (38.8)	
3	12 (3.6)	5 (10.9)	7 (2.4)	
4	32 (9.6)	9 (19.6)	23 (8.0)	
5	14 (4.2)	1 (2.2)	13 (4.5)	
mFS				0.016
1	90 (26.9)	4 (8.7)	86 (29.8)	
2	83 (24.8)	13 (28.3)	70 (24.)	
3	45 (13.4)	6 (13.0)	39 (13.5)	
4	117 (34.9)	23 (50.0)	94 (32.5)	
Hospital course and complications				
Time of extubation				0.012
Ultra-early stage (< 24 h)	267 (79.7)	43 (93.5)	224 (77.5)	
Early stage (≥ 24 h)	68 (20.3)	3 (6.5)	65 (22.5)	
Delayed cerebral ischemia	70 (20.9)	31 (67.4)	39 (13.5)	< 0.001
Hydrocephalus	7 (2.1)	3 (6.5)	4 (1.4)	0.024
Length of hospital stay (days)	13.7 ± 8.1	17.0 ± 8.9	13.2 ± 7.8	0.004
Disability at discharge	2877 (23.5)	27 (65.9)	50 (17.4)	< 0.001
Hospital mortality	7 (2.1)	5 (10.9)	2 (0.7)	< 0.001

Data are given in mean ± SD or median (IQR) and counts (%)

*EF* Extubation failure, *H–H grade* Hunt-Hess grade, *WFNS grade* World Federation of Neurosurgical Societies grade, *mFS* Modified Fisher Scale

In total, 352 aneurysms were repaired in 335 patients. Most of the aneurysms were saccular in morphology (351/352, 99.7%), and 98.9% (347/352) were located in the anterior circulation. The middle cerebral artery is the most common parent artery (118/352, 33.5%), following the anterior communicating artery (114/352, 32.4%). The median (IQR) aneurysm size was 6 (4–8) mm. All of the aneurysms were clipped with microsurgery (Table 2).

**Table 2:** The characteristics of aneurysm

Characteristics	*N* = 352
Morphology	
Saccular	351 (99.7)
Pseudoaneurysm	1 (0.3)
Diameter	6 (4–8)
Circulation	
Anterior	347 (98.9)
Posterior	5 (1.1)
Parent vessel	
ICA	13 (3.7)
ACoA	114 (32.4)
PCoA	84 (23.9)
MCA	118 (33.5)
ACA	18 (5.1)
PICA	5 (1.4)

### Overall perioperative outcomes

The rate of EF was 14% (46/335). Unfortunately, the exact cause for EF often escapes identification. In our data, EF was mainly related to respiratory failure caused by surgical complications such as cerebral infarction, brain edema, intracranial hematoma, and neurological impairment. Only two patients are suffering from upper airway obstruction (laryngeal edema). DCI and acute hydrocephalus were observed in 20.9% (70/335) and 2.1% (7/335) patients, respectively. The average length of hospital stay was 13.7 ± 8.1 days in our cohort. Even with prompt treatment, 23.5% (77/335) of patients were dependent at discharge, and the hospital mortality was 2.1% (7/335).

### Comparisons between aSAH patients with and without extubation failure

No significant difference was observed in medical histories such as smoking, drinking, hypertension, diabetes, hyperlipemia, respiratory disease, coronary disease, prior infarction history, and prior hemorrhage history between aSAH patients with and without EF. We observed that patients with older age and worse disease severity (H–H grade > 2, WFNS grade > 3, and mFS > 2) were more likely to develop EF. Also, EF more frequently appeared in the ultra-early stage compared with the early stage (43/46, 93.5% vs. 3/46, 6.5%). For postoperative complications, 31 (31/46, 67.4%) patients in the EF group and 39 (39/289, 13.5%) patients developed DCI. Hydrocephalus occurred in 3 (3/46, 6.5%) patients in the EF group and 4 (4/289, 1.4%) patients in the non-EF group. Patients with EF tended to have a longer hospital stay, a higher mortality rate, and a worse discharge outcome.

The multivariate model included the following variables: age, time from onset to admission, WFNS grade, and extubation time (Table 3). Patients with an older age had an OR of 1.038 for developing EF (95% CI 1.004–1.073; *p* = 0.028). Additionally, patients with a shorter time from onset to admission had an OR of 0.731 for developing EF (95% CI 0.566–0.943; *p* = 0.016). There was a significant interaction between poor WFNS grade (> 3) (OR 4.309, 95% CI 1.639–11.330; *p* = 0.003) and ultra-early extubation (< 24 h) (OR10.280, 95% CI 2.523–41.884; *p* < 0.001) with the appearance of EF.

**Table 3:** Logistic regression analysis for risk factors of extubation failure

Clinical characteristics	EF	None-EF	Univariable	Multivariable	
	*n* = 46	*n* = 289	*p* value	OR (95%CI)	*p* value
Age	56.0 ± 9.4	53.5 ± 10.5	0.131	1.038 (1.004–1.073)	0.028
Female	25 (54.3)	165 (57.1)	0.727		
Onset to admission (days)	1 (0–2)	2 (0–4)	0.006	0.731 (0.566–0.943)	0.016
Brain herniation	2 (4.3)	8 (2.8)	0.559		
H–H grade > 2	19 (41.3)	83 (28.7)	0.085		
WFNS grade > 3	10 (21.7)	36 (12.5)	0.089	4.309 (1.639–11.330)	0.003
mFS > 2	29 (63.0)	133 (46.0)	0.032		
Aneurysm size (mm)	6 (1–8)	6 (4–8)	0.834		
Smoking	9 (19.6)	50 (17.3)	0.708		
Respiratory disease	1 (2.2)	3 (1.0)	0.510		
Time of extubation < 24 h	3 (6.5)	65 (22.5)	0.012	10.280 (2.523–41.884)	0.001

Data are given in mean ± SD or median (IQR) and counts (%)

*EF* Extubation failure, *H–H grade* Hunt-Hess grade, *WFNS grade* World Federation of Neurosurgical Societies grade, *mF* Modified Fisher Scale

**Fig. 1:**
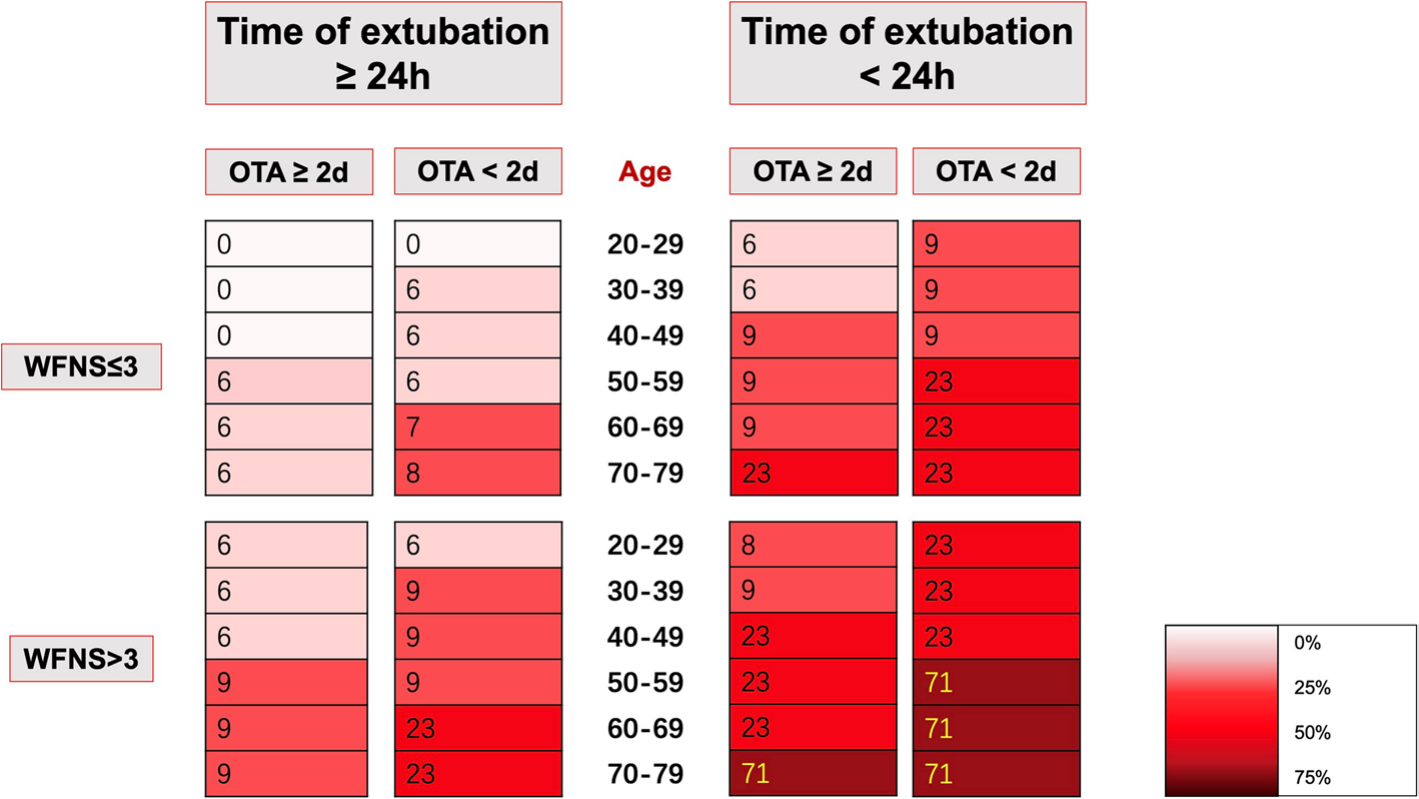
Prediction chart with absolute probabilities (%) of extubation failure in aSAH patients

**Fig. 2:**
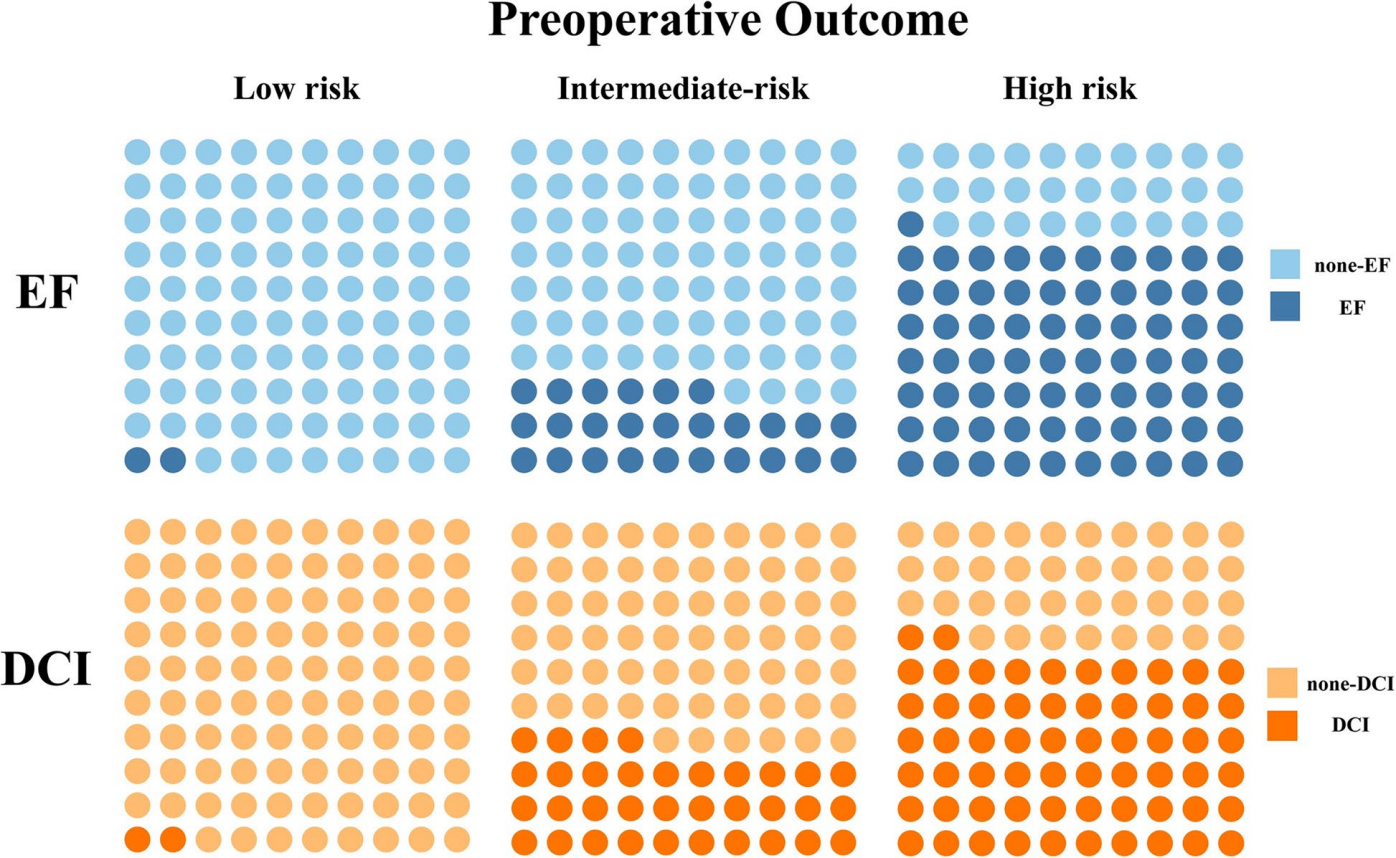
The proportion of patients in different risk groups occurred EF and DCI

We translated the regression coefficients into a score chart presented in Table S1. This score can be combined with Figure S1 to obtain the predicted probabilities for individual persons. Figure 1 shows a risk chart with estimated probabilities of developing EF after microsurgery in aSAH patients to age, onset to admission time, disease severity, and time of extubation. Based on the result of the multivariable model, age ≥ 56, onset to admission time < 2 days, WFNS grade > 3, and extubation time < 24 h were defined as the four main factors closely associated with EF. We stratified the patients into three groups according to the presence of four risk factors: the low-risk group has no risk factors, the intermediate-risk group only has one risk factor, and the high-risk group has two or more risk factors. Compared with other groups, the proportion of patients who had EF and DCI was higher in the high-risk group (Fig. 2).

### Subgroup analysis in propensity score-matched cohort

The variances of included raw variables between ultra-early and early extubation groups were significantly different (Table S2 and Fig. S2). PS for having early extubation was generated using age, sex, herniation, preoperative H–H grade, preoperative WFNS grade, preoperative mFS, and preoperative mRS grade. Using propensity score matching, 54 patients who underwent early extubation were matched to patients performed with ultra-early extubation. The variance between treatment groups after matching was not different (Fig. S2). A love plot showed that all characteristics achieved the desirable cut-off of standardized mean differences < 0.1 in the matched dataset (Fig. S3). In the matched pairs, there was a trend toward a higher incidence of EF in ultra-early extubation patients (16/54, 29.6%) than in patients with early extubation (3/54, 5.6%). In the subgroup analysis by age, onset to admission time, and WFNS grade, ultra-early extubation was associated with a higher incidence of EF (Table 4). For patients with WFNS grade > 3, ultra-early extubation showed a trend to significantly increase the the occurrence of DCI.

**Table 4:** Perioperative outcomes in the risk factor subgroups in the propensity score-matched cases

	Ultra-early stage extubation	Early stage extubation	*p* value
Total	54	54	
Extubation failure	16 (29.6)	3 (5.6)	< 0.001^a^
Delayed cerebral ischemia	15 (27.8)	15 (27.8)	1.000^a^
Hospital mortality	2 (3.7)	3 (5.6)	1.000^a^
Age ≥ 56	21	29	
Extubation failure	9 (42.9)	2 (6.9)	0.002
Delayed cerebral ischemia	7 (33.3)	7 (24.1)	0.475
Hospital mortality	2 (9.5)	2 6.9)	0.735
Onset to admission < 2 day	26	23	
Extubation failure	10 (38.5)	1 (4.3)	0.004
Delayed cerebral ischemia	11 (42.3)	12 (52.2)	0.490
Hospital mortality	1 (3.8)	2 (8.7)	0.480
WFNS grade > 3	13	13	
Extubation failure	9 (69.2)	0 (0)	< 0.001
Delayed cerebral ischemia	9 (69.2)	4 (30.8)	0.050
Hospital mortality	2 (15.4)	0 (0)	0.141

Data are given in counts (%)

*WFNS grade* World Federation of Neurosurgical Societies grade

^a^McNemar test

## Discussion

This study demonstrated that retaining the endotracheal intubation is commonly needed in aSAH patients. We showed that clinical findings for disease severity evaluated within 24 h of admission could predict patients at higher risk of EF. Furthermore, aSAH patients with older age, onset time < 2 days, and WFNS > 3 may not suggest extubation in 24 h; prolonged intubation might benefit them. There is a lot to guide clinicians in the intubation management of neuroscience intensive care unit patients [19–21]. In general, the normal ICU weaning patterns and extubation algorithms may not be simply applied to neuro-ICU patients, since they often need special care to avoid or minimize secondary brain damage. There is an elevated risk for secondary brain injury when extubation is not carefully planned and patients are not checked for reintubation risks like difficult airway situations. This information can be useful for the clinician in the communication with relatives and the care of critically ill aSAH patients.

In the present study, EF occurred with a relatively low ratio of 0.14, compared with the rates between 0.11 and 0.44 reported in the previously published studies [8, 13, 22–24]. Advanced age has been proven to be an important factor associated with an increased risk of EF. The incidence of acute respiratory failure of any cause is more than three times higher in patients aged > 65 than in the younger population [25, 26]. Previous studies reported that 20% to 35% of elderly patients were reintubated within 48 to 72 h after extubation [27, 28]. A prospective cohort study also indicated that extubation success was predicted by younger age, presence of cough, and negative fluid balance, rather than Glasgow Coma Scale scores at extubation [29]. Our data also suggested that EF patients are characterized by older age. This may be because advanced age and frailty are associated with a higher frequency of comorbidities (such as pulmonary and cardiovascular diseases) and loss of reserve across multiple physiologic systems making these patients susceptible to adverse events [30]. Our study found that the causes of extubation failure included infarction-related complications (23/46,50.0%), cerebral edema (6/46, 13.0%), unconsciousness (5/46, 10.9%), hypoxemia (4/46, 8.7%), pulmonary infection (2/46, 4.3%), laryngeal edema (2/46, 4.3%), pulmonary embolism (2/46, 4.3%), and rebleeding (2/46, 4.3%). The deterioration of neurological function is the main reason for the EF, and the deterioration of neurological function is mainly related to the severity of SAH, which further confirms the conclusion of our study that tracheal intubation can be appropriately retained for patients with risk factors.

Considering weaning failure is associated with a poor prognosis, reintubation may elevate mortality. Extubation determination for critically ill and elderly patients may require more caution. A spontaneous breathing trial needs to be prolonged in those patients.

A higher WFNS grade implied a decreased level of consciousness and focal neurologic deficits resulting in inadequate protection reflexes and risk for aspiration. Importantly, the WFNS grade is traditionally associated with a higher risk of DCI and poor outcomes, which may be associated with prolonged intubation [10, 31]. Moreover, the severity of the SAH-associated intracranial pathology increased the risk of respiratory failure. Historically, clipping between days 5 to 10 is considered the worst period for neurosurgery [32, 33]. Results of several studies suggested that patients undergoing early surgery (days 0–3) tended to have the best prognosis [34–36]. However, patients undergoing the early surgery (< 2 days) tended to have EF in this study. This may partially be explainable by an exacerbation of the bleeding with associated intracranial pathology. Even though the microsurgery repaired the aneurysm, damage to the brain tissue from the bleeding actually continued over time. Thus, incorrect assessment of neurological status after early surgery may be an imperative cause of EF.

Patients with prior cerebral infarction were more prone to post-extubation dysphagia, which became a growing concern as a major risk factor for EF and a significant contributor to poor patient outcomes [37]. However, our study showed that prior cerebral infarction was not significantly associated with EF, as well as other medical histories such as hypertension, diabetes, hyperlipemia, respiratory disease, coronary disease, and prior hemorrhage. It somewhat indicated that the occurrence of EF mainly depends on the neurological status rather than the previous medical history in aSAH patients.

According to the previous study [6–8, 37], reintubated within 48 to 72 h was associated with a longer hospital stay and a higher mortality rate. Obviously, our patients with aSAH and EF had a longer need for mechanical ventilation than patients after successful extubation. DCI is a clinical syndrome of focal neurological deficits or a radiologically confirmed infarction that occurs in one-third of patients after aSAH and is caused by an interaction of multiple vascular and neural changes, including impaired autoregulation and vasospasm of intracranial arteries [38]. It is a well-known contributing factor to mortality and long-term handicaps after aSAH. Also, the acute hydrocephalus after the aSAH led to unfavored neurological function and poor prognosis. Interestingly, patients with EF were more likely to develop DCI and acute hydrocephalus in this study, which might indicate that EF is responsible for the worsening of the outcome. Thus, the fact that EF seems to be associated with a poor prognosis could be an important factor that might be helpful for clinicians in improving the patient’s treatment.

The optimal extubation timing of aSAH patients is unknown once the standard criteria to consider extubation are met. On the other hand, there is no evidence of postponing extubation to prevent a patient from a poor outcome. Our institution keeps a more positive attitude towards extubation. After surgery, the tracheal intubation is usually removed within 24 h for patients without obvious disturbance of consciousness and impaired brainstem function. Using PS matching to reduce the baseline difference between the two groups, our data indicated that the EF majority occurs in the ultra-early group. Further subgroup analysis demonstrated that ultra-early extubation significantly increases the risk of EF in high-risk groups. However, our data is not abundant enough to provide an exact extubation time for all aSAH patients. It seems like prolonged intubation may be beneficial for aSAH patients with older age, more acute onset, and worse disease severity. In the long run, rather than dogmatic reductions in unnecessary medical measures, reasonable reliance on adjuvant medical measures may lead to a better patient outcome.

Limitations of our data include patient select bias, limited data on the acute physiology score, constraints inherent in any retrospective study, and lack of data relative to respiratory failure, which is beyond the scope and capabilities of a case–control analysis. A prospective randomized trial would need to be conducted to establish the optimal extubation time in a given population. However, a randomized trial of extubation time in critically ill aSAH patients would be unethical.

## Conclusions

These data provide further evidence that older aSAH patients with onset to admission time < 2 days and WFNS grade > 3 have a high risk of developing EF, which is amplified by the ultra-early extubation. Moreover, in patients with two or more risk factors, a prolonged intubation recommendation requires consideration to avoid the EF. Ultimately, physicians must weigh the potential risks of postponing extubation and EF when making the extubation decision.

## Supplementary Information

Supplementary Material 1.

## Data Availability

All original data are available upon reasonable request to the corresponding authors.

## References

[1] Yang J, Lu J, Li R, et al. Application of intracranial pressure-directed therapy on delayed cerebral ischemia after aneurysmal subarachnoid hemorrhage. Front Aging Neurosci. 2022;14:831994. 35360218 10.3389/fnagi.2022.831994 PMC8964287

[2] Chung DY, Abdalkader M, Nguyen TN. Aneurysmal subarachnoid hemorrhage. Neurol Clin. 2021;39(2):419–42. 33896527 10.1016/j.ncl.2021.02.006 PMC8147706

[3] Florez WA, Garcia-Ballestas E, Deora H, et al. Intracranial hypertension in patients with aneurysmal subarachnoid hemorrhage: a systematic review and meta-analysis. Neurosurg Rev. 2021;44(1):203–11. 32008128 10.1007/s10143-020-01248-9

[4] Wojak JF, Ditz C, Abusamha A, et al. The Impact of Extubation Failure in Patients with Good-Grade Subarachnoid Hemorrhage. World Neurosurg. 2018;117:e335–40. 29908380 10.1016/j.wneu.2018.06.027

[5] Rass V, Ianosi BA, Lindlbauer M, et al. Factors associated with prolonged mechanical ventilation in patients with subarachnoid hemorrhage-the RAISE Score. Crit Care Med. 2022;50(1):103–13. 34259444 10.1097/CCM.0000000000005189

[6] Thille AW, Richard JC, Brochard L. The decision to extubate in the intensive care unit. Am J Respir Crit Care Med. 2013;187(12):1294–302. 23641924 10.1164/rccm.201208-1523CI

[7] Vallverdu I, Calaf N, Subirana M, Net A, Benito S, Mancebo J. Clinical characteristics, respiratory functional parameters, and outcome of a two-hour T-piece trial in patients weaning from mechanical ventilation. Am J Respir Crit Care Med. 1998;158(6):1855–62. 9847278 10.1164/ajrccm.158.6.9712135

[8] Rishi MA, Kashyap R, Wilson G, Schenck L, Hocker S. Association of extubation failure and functional outcomes in patients with acute neurologic illness. Neurocrit Care. 2016;24(2):217–25. 26215402 10.1007/s12028-015-0156-3

[9] Molyneux AJ, Kerr RSC, Yu L-M, et al. International subarachnoid aneurysm trial (ISAT) of neurosurgical clipping versus endovascular coiling in 2143 patients with ruptured intracranial aneurysms: a randomised comparison of effects on survival, dependency, seizures, rebleeding, subgroups, and aneurysm occlusion. The Lancet. 2005;366(9488):809–17. 10.1016/S0140-6736(05)67214-5 16139655

[10] Li R, Lin F, Chen Y, et al. In-hospital complication-related risk factors for discharge and 90-day outcomes in patients with aneurysmal subarachnoid hemorrhage after surgical clipping and endovascular coiling: a propensity score-matched analysis. J Neurosurg. 2021;137(2):381–92. 34972088 10.3171/2021.10.JNS211484

[11] Connolly ES Jr, Rabinstein AA, Carhuapoma JR, et al. Guidelines for the management of aneurysmal subarachnoid hemorrhage: a guideline for healthcare professionals from the American Heart Association/american Stroke Association. Stroke. 2012;43(6):1711–37. 22556195 10.1161/STR.0b013e3182587839

[12] Torrini F, Gendreau S, Morel J, et al. Prediction of extubation outcome in critically ill patients: a systematic review and meta-analysis. Crit Care. 2021;25(1):391. 34782003 10.1186/s13054-021-03802-3 PMC8591441

[13] Guru PK, Singh TD, Pedavally S, Rabinstein AA, Hocker S. Predictors of extubation success in patients with posterior fossa strokes. Neurocrit Care. 2016;25(1):117–27. 26886009 10.1007/s12028-016-0249-7

[14] Robba C, Poole D, McNett M, et al. Mechanical ventilation in patients with acute brain injury: recommendations of the European Society of Intensive Care Medicine consensus. Intensive Care Med. 2020;46(12):2397–410. 33175276 10.1007/s00134-020-06283-0 PMC7655906

[15] Report of World Federation of Neurological Surgeons Committee on a Universal Subarachnoid Hemorrhage Grading Scale. J Neurosurg. 1988;68(6):985–6. 10.3171/jns.1988.68.6.0985 3131498

[16] Bor-Seng-Shu E, Paiva WS, Figueiredo EG, et al. Posttraumatic refractory intracranial hypertension and brain herniation syndrome: cerebral hemodynamic assessment before decompressive craniectomy. Biomed Res Int. 2013;1:750809. 10.1155/2013/750809 PMC3860083 24377095

[17] Bullock MR, Chesnut R, Ghajar J, et al. Surgical management of traumatic parenchymal lesions. Neurosurgery. 2006;58(3 Suppl):S25-46. 16540746 10.1227/01.NEU.0000210365.36914.E3

[18] Vergouwen MD, Vermeulen M, van Gijn J, et al. Definition of delayed cerebral ischemia after aneurysmal subarachnoid hemorrhage as an outcome event in clinical trials and observational studies: proposal of a multidisciplinary research group. Stroke. 2010;41(10):2391–5. 20798370 10.1161/STROKEAHA.110.589275

[19] Godet T, Chabanne R, Marin J, et al. Extubation failure in brain-injured patients: risk factors and development of a prediction score in a preliminary prospective cohort study. Anesthesiology. 2017;126(1):104–14. 27749290 10.1097/ALN.0000000000001379

[20] Bosel J. Who is safe to extubate in the neuroscience intensive care unit? Semin Respir Crit Care Med. 2017;38(6):830–9. 29262440 10.1055/s-0037-1608773

[21] Battaglini D, Siwicka Gieroba D, Brunetti I, et al. Mechanical ventilation in neurocritical care setting: A clinical approach. Best Pract Res Clin Anaesthesiol. 2021;35(2):207–20. 34030805 10.1016/j.bpa.2020.09.001

[22] Karanjia N, Nordquist D, Stevens R, Nyquist P. A clinical description of extubation failure in patients with primary brain injury. Neurocrit Care. 2011;15(1):4–12. 21394542 10.1007/s12028-011-9528-5

[23] Anderson CD, Bartscher JF, Scripko PD, et al. Neurologic examination and extubation outcome in the neurocritical care unit. Neurocrit Care. 2011;15(3):490–7. 20428967 10.1007/s12028-010-9369-7

[24] Steidl C, Bosel J, Suntrup-Krueger S, et al. Tracheostomy, extubation, reintubation: airway management decisions in intubated stroke patients. Cerebrovasc Dis. 2017;44(1–2):1–9. 28395275 10.1159/000471892

[25] Behrendt CE. Acute respiratory failure in the United States: incidence and 31-day survival. Chest. 2000;118(4):1100–5. 11035684 10.1378/chest.118.4.1100

[26] Suraseranivong R, Krairit O, Theerawit P, Sutherasan Y. Association between age-related factors and extubation failure in elderly patients. PLoS ONE. 2018;13(11):e0207628. 30458035 10.1371/journal.pone.0207628 PMC6245685

[27] Su KC, Tsai CC, Chou KT, et al. Spontaneous breathing trial needs to be prolonged in critically ill and older patients requiring mechanical ventilation. J Crit Care. 2012;27(3):324 e1-7. 21798702 10.1016/j.jcrc.2011.06.002

[28] El Solh AA, Bhat A, Gunen H, Berbary E. Extubation failure in the elderly. Respir Med. 2004;98(7):661–8. 15250233 10.1016/j.rmed.2003.12.010

[29] McCredie VA, Ferguson ND, Pinto RL, et al. Airway management strategies for brain-injured patients meeting standard criteria to consider extubation. A prospective cohort study. Ann Am Thorac Soc. 2017;14(1):85–93. 27870576 10.1513/AnnalsATS.201608-620OC

[30] Fried LP, Ferrucci L, Darer J, Williamson JD, Anderson G. Untangling the concepts of disability, frailty, and comorbidity: implications for improved targeting and care. J Gerontol A Biol Sci Med Sci. 2004;59(3):255–63. 15031310 10.1093/gerona/59.3.m255

[31] Li R, Lin F, Chen Y, et al. A 90-day prognostic model based on the early brain injury indicators after aneurysmal subarachnoid hemorrhage: the TAPS Score. Transl Stroke Res. 2022;14(2):200–10. 35567655 10.1007/s12975-022-01033-4

[32] Kassell NF, Torner JC, Jane JA, Haley EC Jr, Adams HP. The International Cooperative Study on the Timing of Aneurysm Surgery. Part 2: Surgical results. J Neurosurg. 1990;73(1):37–47. 2191091 10.3171/jns.1990.73.1.0037

[33] Ohman J, Heiskanen O. Timing of operation for ruptured supratentorial aneurysms: a prospective randomized study. J Neurosurg. 1989;70(1):55–60. 2909689 10.3171/jns.1989.70.1.0055

[34] Ross N, Hutchinson PJ, Seeley H, Kirkpatrick PJ. Timing of surgery for supratentorial aneurysmal subarachnoid haemorrhage: report of a prospective study. J Neurol Neurosurg Psychiatry. 2002;72(4):480–4. 11909907 10.1136/jnnp.72.4.480 PMC1737846

[35] Whitfield PC, Kirkpatrick PJ. Timing of surgery for aneurysmal subarachnoid haemorrhage. Cochrane Database Syst Rev. 2001;2:CD001697. 10.1002/14651858.CD001697 11405999

[36] de Gans K, Nieuwkamp DJ, Rinkel GJ, Algra A. Timing of aneurysm surgery in subarachnoid hemorrhage: a systematic review of the literature. Neurosurgery. 2002;50(2):336–40. 11844269 10.1097/00006123-200202000-00018

[37] Suntrup-Krueger S, Schmidt S, Warnecke T, et al. Extubation readiness in critically ill stroke patients. Stroke. 2019;50(8):1981–8. 31280655 10.1161/STROKEAHA.118.024643

[38] Rass V, Helbok R. How to diagnose delayed cerebral ischaemia and symptomatic vasospasm and prevent cerebral infarction in patients with subarachnoid haemorrhage. Curr Opin Crit Care. 2021;27(2):103–14. 33405414 10.1097/MCC.0000000000000798

