# Supplementary material for: Extubation timing and risk of extubation failure in aneurysmal subarachnoid hemorrhage patients
Source: Chin Neurosurg J. 2024 Nov 20;10:32. doi: 10.1186/s41016-024-00384-1 (PMC11577864; doi:10.1186/s41016-024-00384-1)
Supplement: Supplementary file 1 — Supplementary Material 1. [file 41016_2024_384_MOESM1_ESM.docx]

Table S1 Regression equations of multivariable models

| Regression equation model based on person characteristics |
| --- |
| -5.912+ 0.331065*Age+ 1.002198*Onset to Admission <2d+ 1.423189*WFNS grade >3+ 2.291073*Ultra-early Extubation |

Table S2. Baseline characteristics before and after the propensity score-matched

|  | Unmatched | | | Matched | | |
| --- | --- | --- | --- | --- | --- | --- |
|  | Early | Ultra-early |  | Early | Ultra-early |  |
|  | 68 | 267 | p Value | 54 | 54 | p Value |
| Age,years | 58.3 ± 9.5 | 52.7 ± 10.3 | < 0.001 | 57.1 ± 9.3 | 54.7 ± 8.9 | 0.295* |
| Sex |  |  | 0.505 |  |  | 0.350† |
| Female | 41 (60.3) | 149 (55.8) |  | 30 (55.6) | 32 (59.3) |  |
| Male | 27 (39.7) | 118 (44.2) |  | 24 (44.4) | 22 (40.7) |  |
| **Comorbidities** |  |  |  |  |  |  |
| Smoking | 12 (17.6) | 47 (17.6) | 0.993 | 10 (18.5) | 11 (20.4) | 1.000† |
| Drinking | 7 (10.3) | 24 (9.0) | 0.740 | 5 (9.3) | 5 (9.3) | 1.000† |
| Hypertension | 37 (54.5) | 119 (44.6) | 0.146 | 31 (57.4) | 29 (53.7) | 0.845† |
| Diabetes | 6 (8.8) | 18 (6.7) | 0.552 | 5 (9.3) | 8 (14.8) | 0.549† |
| Hyperlipemia | 2 (2.9) | 7 (2.6) | 0.884 | 1 (1.9) | 3 (5.6) | 0.625† |
| Respiratory disease | 1 (1.5) | 3 (1.1) | 0.814 | 1 (1.9) | 1 (1.9) | 1.000† |
| Coronary disease | 3 (4.4) | 7 (2.6) | 0.439 | 2 (3.7) | 2 (3.7) | 1.000† |
| Prior infarction | 3 (4.4) | 19 (7.1) | 0.422 | 3 (5.6) | 8 (14.8) | 0.227† |
| Prior hemorrhage | 1 (1.5) | 1 (0.4) | 0.295 | 1 (1.9) | 0 (0) | 1.000† |
| **Admission characteristics** |  |  |  |  |  |  |
| Onset to admission (d) | 2 (1-4) | 2 (1-3) | 0.036 | 2 (1-5) | 2 (1-3) | 0.101§ |
| Herniation | 8 (11.8) | 2 (0.7) | < 0.001 | 2 (3.7) | 2 (3.7) | 1.000† |
| H-H grade |  |  | 0.041 |  |  | 0.861‡ |
| 1 | 5 (7.4) | 35 (13.1) |  | 5 (9.3) | 3 (5.6) |  |
| 2 | 33 (48.5) | 160 (59.9) |  | 26 (48.1) | 29 (53.7) |  |
| 3 | 1 (1.5) | 4 (1.5) |  | 0 (0) | 2 (3.7) |  |
| 4 | 29 (42.6) | 68 (25.5) |  | 23 (42.6) | 20 (37.0) |  |
| WFNS grade |  |  | < 0.001 |  |  | 0.423‡ |
| 1 | 14 (20.6) | 128 (47.9) |  | 14 (25.9) | 15 (27.8) |  |
| 2 | 23 (33.8) | 112 (41.9) |  | 23 (42.6) | 24 (44.4) |  |
| 3 | 4 (5.9) | 8 (3.0) |  | 4 (7.4) | 2 (3.7) |  |
| 4 | 15 (22.1) | 17 (6.4) |  | 10 (18.5) | 12 (22.2) |  |
| 5 | 12 (17.6) | 2 (0.7) |  | 3 (5.6) | 1 (1.9) |  |
| mFS |  |  | 0.001 |  |  | 0.269‡ |
| 1 | 8 (11.8) | 82 (30.7) |  | 7 (13.0) | 15 (27.8) |  |
| 2 | 16 (23.5) | 67 (25.1) |  | 15 (27.8) | 9 (16.7) |  |
| 3 | 7 (10.3) | 38 (14.2) |  | 6 (11.1) | 6 (11.1) |  |
| 4 | 37 (54.4) | 80 (30.0) |  | 26 (48.1) | 24 (44.4) |  |
| mRS grade |  |  | < 0.001 |  |  | 0.275‡ |
| 1 | 32 (47.1) | 238 (89.1) |  | 32 (59.3) | 38 (70.4) |  |
| 2 | 21 (30.9) | 20 (7.5) |  | 16 (29.6) | 7 (13.0) |  |
| 3 | 6 (8.8) | 5 (1.9) |  | 1 (1.9) | 5 (9.3) |  |
| 4 | 6 (8.8) | 3 (1.1) |  | 3 (5.6) | 3 (5.6) |  |
| 5 | 3 (4.4) | 1 (0.4) |  | 2 (3.7) | 1 (1.9) |  |

Data are given in mean ± SD or median (IQR) and counts (%).

* Wilcoxon test.

† McNemar test.

‡ Marginal homogeneity test.

§ Mann-Whitney U-test.

Figure S1. Predicted probability of EF after microsurgery in aSAH patients on the prediction score


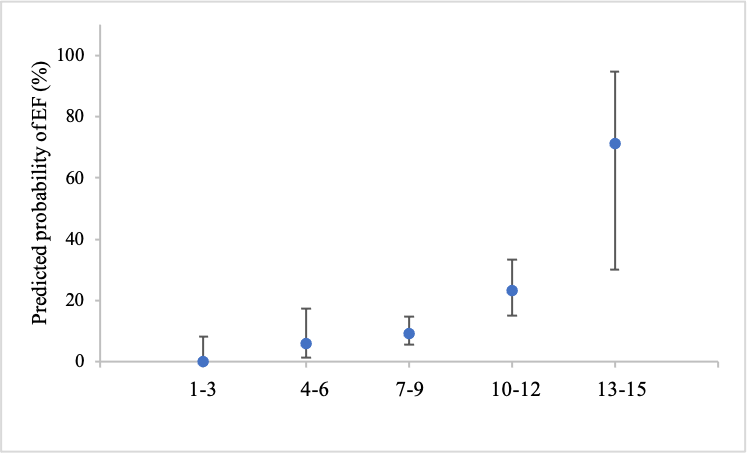


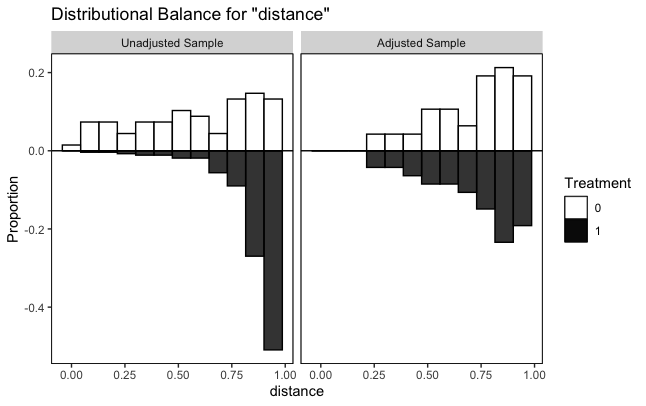
Figure S2. Standardized mean differences of included variables in the raw dataset and matched dataset

Standardized mean differences of included variables in the raw dataset and matched dataset were calculated and compared.


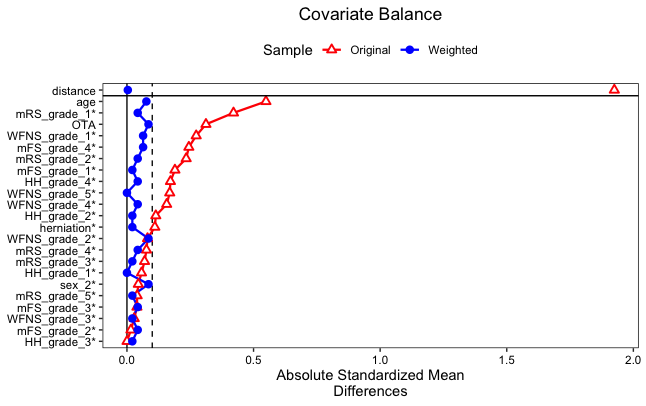
Figure S3. Absolute standardized mean differences of included variables in the raw dataset and matched dataset

All characteristics achieved the desirable cut-off of standardized mean differences <0.1 in the matched dataset.
